# Cytokine modulation by etanercept ameliorates metabolic syndrome and its related complications induced in rats administered a high-fat high-fructose diet

**DOI:** 10.1038/s41598-022-24593-9

**Published:** 2022-11-23

**Authors:** Noha F. Hassan, Azza H. Hassan, Mona R. El-Ansary

**Affiliations:** 1 Department of Pharmacology and Toxicology, Faculty of Pharmacy, Modern University for Technology and Information, Cairo, Egypt; 2 Department of Pathology, Faculty of Veterinary Medicine, Cairo University, Cairo, Egypt; 3 Department of Biochemistry, Faculty of Pharmacy, Modern University for Technology and Information, Cairo, Egypt

**Keywords:** Gastrointestinal diseases, Biochemistry

## Abstract

The aim of the present study was to investigate the effect of etanercept (ETA)—an anti-tumor necrosis factor α (TNF-α) monoclonal antibody—on metabolic disorders such as obesity, hypertension, dyslipidemia, and insulin resistance associated with the metabolic syndrome (MS). MS was induced in rats via high-fat high-fructose (HFHF) administration for 8 weeks. Rats were divided into three groups: negative control, HFHF model, and ETA-treated groups [HFHF + ETA (0.8 mg/kg/twice weekly, subcutaneously) administered in the last 4 weeks]. ETA effectively diminished the prominent features of MS via a significant reduction in the percent body weight gain along with the modulation of adipokine levels, resulting in a significant elevation of serum adiponectin consistent with TNF-α and serum leptin level normalization. Moreover, ETA enhanced dyslipidemia and the elevated blood pressure. ETA managed the prominent features of MS and its associated complications via the downregulation of the hepatic inflammatory pathway that induces nonalcoholic steatohepatitis (NASH)—from the expression of Toll-like receptor 4, nuclear factor kappa B, and TNF-α until that of transforming growth factor—in addition to significant improvements in glucose utilization, insulin sensitivity, and liver function parameter activity and histopathological examination. ETA was effective for the treatment of all prominent features of MS and its associated complications, such as type II diabetes mellitus and NASH.

## Introduction

Considering the significant increase in the cases of metabolic syndrome (MS) globally, with an increasing prevalence rate of 20–30%^1^, MS has become the most epidemically pathological condition, with > 1 billion individuals experiencing MS, particularly in the Middle East^2,3^. This fact is attributable to the widespread well-defined symptoms of MS, such as obesity, hepatic damage, insulin resistance (IR), dyslipidemia, and hypertension, as well as to these symptoms being the leading causes of metabolic complications, such as cardiovascular disorders, type 2 diabetes mellitus, and nonalcoholic fatty liver diseases (NAFLD); this fact renders MS a new public health concern globally^1,4,5^.

Recently, studies have combined dietary models to induce MS and investigate possible interventions owing to the scarcity of animal models that are entirely analogous to human MS pathogenesis^1,4^.

Significant uptake of high-fat, high-fructose (HFHF) in the diet is the first marked step of MS at the pathogenesis level. Excess adipogenesis and lipogenesis stimulate the elevated secretions of adipocytokines, such as leptin overproduction, thereby developing leptin resistance and eventually inducing obesity^1,5^. The production of MS cytokines during adipocyte dysfunction fluctuates with the significant increase in tumor necrosis factor-α (TNF-α) expression, and hypoadiponectinemia can lead to inflammation, IR, hypertension, and hepatic steatosis^6,7^. Collectively, these adipose tissue secretomes lead to further systemic complications associated with MS^8^, such as nonalcoholic steatohepatitis (NASH), which evolves and progresses owing to the activation of the hepatic Toll-like receptor 4 (TLR4)/nuclear factor-κB (NF-κB)/TNF-α/transforming growth factor-β1 (TGF-β1) signaling pathway by endotoxemia resulting from an excessive HFHF dietary intake^4,9,10^.

To date, there is a crucial need for MS-approved pharmacological therapies. Therefore, recent studies have focused on developing novel effective treatments targeting the downregulation of different molecular mechanisms that motivate MS and its associated metabolic disturbances^2,9^.

Accordingly, the present study assessed the efficacy of etanercept (ETA) for treating MS and its related complications using an HFHF diet animal model. ETA, a recombinant human soluble TNF-α receptor protein, is a potent TNF-α antagonist that reduces TNF-α activity by competitively blocking its binding with its receptor^11^. To date, ETA is mainly used to treat TNF-α-related disorders, such as rheumatoid arthritis, Crohn’s disease, ankylosing spondylitis, and psoriasis^12^. Previous studies have indicated that TNF-α is the key inducer of the well-defined features of MS, such as obesity, impaired glucose utilization, IR, dyslipidemia, and hepatotoxicity^13,14^. Although ETA was withdrawn from the clinical trial which was investigating its therapeutic effects in the treatment of heart failure as during the trial period ETA did not show therapeutic effectiveness^15^, it is worth noting that, clinical trials are still undergoing investigation of the efficacy and safety of ETA in treating different metabolic disorders in which ETA showed promising results not only in clinical trials that targeted metabolic disorders accompanied by autoimmune diseases^16–19^ but also, in clinical trials targeting patients suffering from metabolic diseases only^20–22^. Besides, several articles concluded that ETA will be one of the future treatments that still need further studies either in type 2 diabetes^23^ or even in chronic heart failure^24,25^. All that opened the door for further experimental studies to be held for additional investigations of ETA in the treatment of different metabolic diseases^14,26–28^. Regarding its safety, all the previously mentioned clinical and experimental studies confirmed that after close monitoring of the patients who participated in the trial, ETA was safe, effective, and tolerable within the used dose and trial period that was indicated in each study. Though, anti-cytokine remedies aren’t devoid of side effects; thus, benefits should be carefully weighed against risks concerned with the use of these therapies which still need further studies.

Hence, using potent TNF-α antagonists like ETA could effectively manage MS by preventing its progression via the downregulation of the NF-κB/TNF-α/TGF-β1 signaling pathway and treatment of adipocyte dysfunction, impaired glucose utilization, dyslipidemia, hypertension, and obesity as investigated and confirmed in the current study.

## Results

### ETA counteracts HFHF-induced obesity

**Figure 1:**
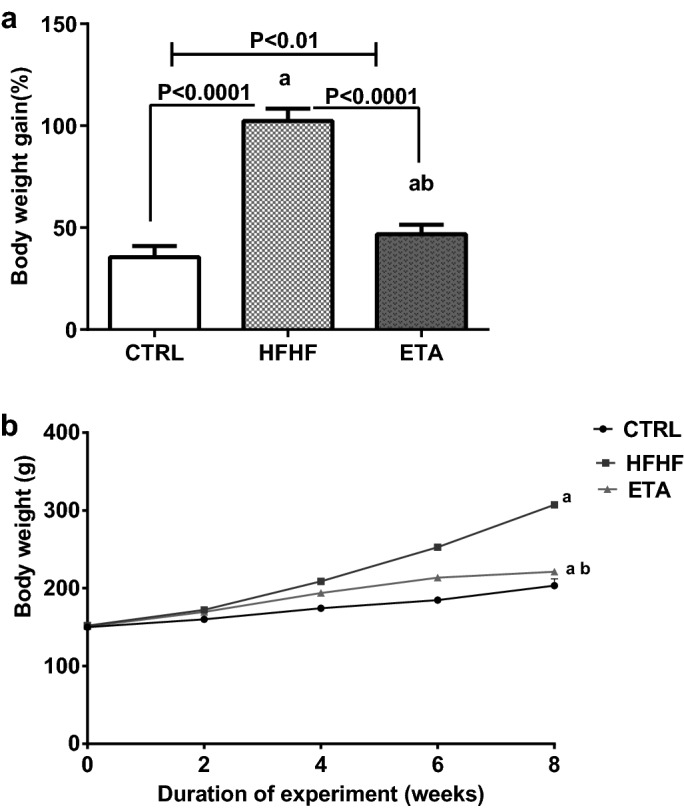
ETA counteracts HFHF-induced body weight gain. Comparison data from the HFHF-fed rats and standard chow-fed rats (CTRL) are shown: (**a**) percent body weight gain and (**b**) gradual increase in body weight. Data are presented as the mean ± SD values and are representative of a single independent experiment (*n* = 6). Statistical analysis was performed using one-way ANOVA, followed by Tukey’s multiple comparison tests. ^a^Significantly different from the normal control group. Differences were considered significant if the p-value was < 0.05. *CTRL* control, *ETA* etanercept, *HFHF* high-fat high-fructose diet.

Compared with the HFHF group, in the ETA group, ETA counteracted any increase in body weight and significantly reduced the percent body weight gain by 54.3% (Fig. 1a). Animals in the HFHF group showed an increase in body weight by 188.4% compared with the normal increase in body weight in the normal control group; moreover, a gradual increase in the mean body weight was observed during the 8-week experimental period in all groups (Fig. 1b).

### ETA attenuates HFHF-induced adipocyte dysfunction

**Figure 2:**
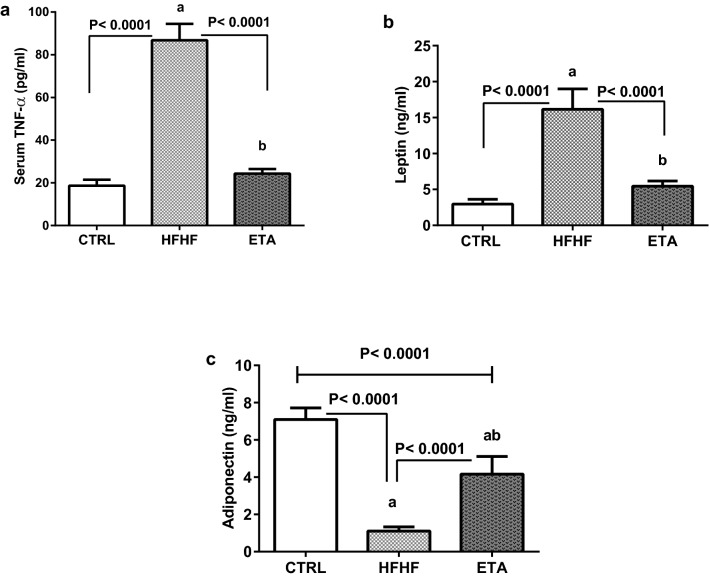
ETA attenuates HFHF-induced adipocyte dysfunction. Comparison data from the HFHF-fed rats and standard chow-fed rats (CTRL) are shown: (**a**) serum TNF-α level, (**b**) serum leptin level, and (**c**) serum adiponectin level. Data are presented as the mean ± SD values and are representative of a single independent experiment (*n* = 6). Statistical analysis was performed using one-way ANOVA, followed by Tukey’s multiple comparison tests. ^a^Significantly different from the normal control group. ^b^Significantly different from the HFHF group. Differences were considered significant if the p-value was < 0.05. *CTRL* control, *ETA* etanercept, *HFHF* high-fat high-fructose diet, *TNF-α* tumor necrosis factor-alpha.

Feeding rats with an HFHF diet caused severe adipocyte dysfunction resulting in a significant augmentation of serum TNF-α and leptin levels (365% and 446.3%, respectively) compared with the control group (Fig. 2a,b). Moreover, adiponectin serum levels showed a significant decline of 84.5% compared with the control group (Fig. 2c).

Treatment with ETA could effectively neutralize the significant elevation of serum TNF-α and leptin levels (Fig. 2a,b). Besides, hypoadiponectinemia recovered by 279% compared with the model group (Fig. 2c).

### ETA attenuates HFHF-induced hypertension

**Figure 3:**
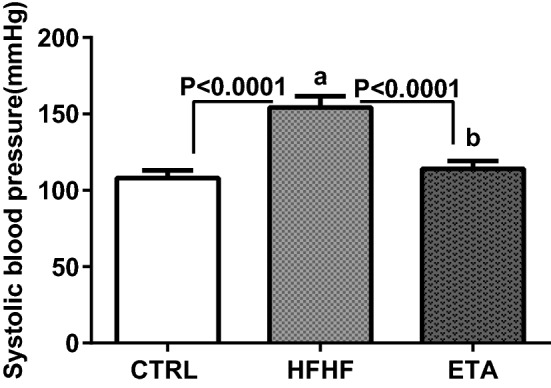
ETA attenuates HFHF-induced systolic blood pressure elevation. Data from the HFHF-fed rats and standard chow-fed rats (CTRL) are shown. Data are presented as the mean ± SD values and are representative of a single independent experiment (*n* = 6). Statistical analysis was performed using one-way ANOVA, followed by Tukey’s multiple comparison tests. ^a^Significantly different from the normal control group. ^b^Significantly different from the HFHF group. Differences were considered significant if the p-value was < 0.05. *CTRL* control, *ETA* etanercept, *HFHF* high-fat high-fructose diet, *SBP* systolic blood pressure.

Rats in the HFHF group showed a significant increase in systolic blood pressure (SBP) by 42.8% compared with those in the normal control group. Treatment with ETA alleviated hypertension as indicated by normal SBP levels (Fig. 3).

### ETA ameliorates HFHF-induced IR

**Figure 4:**
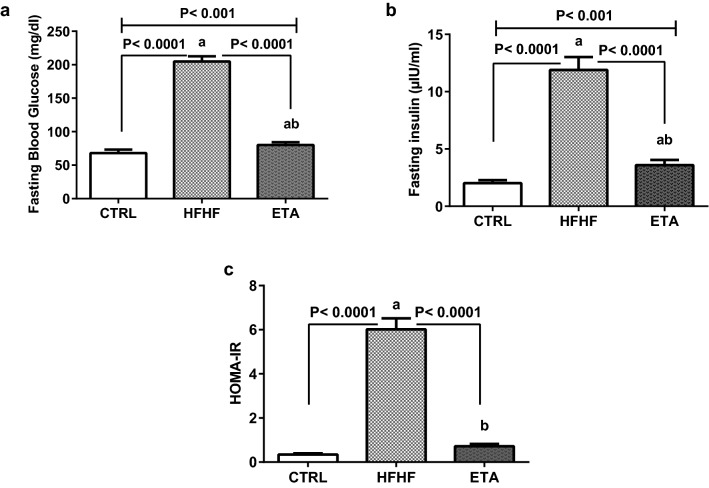
ETA ameliorates HFHF-induced IR. Comparison data from the HFHF-fed rats and standard chow-fed rats (CTRL) are shown: (**a**) fasting blood glucose level, (**b**) serum fasting insulin level, and (**c**) HOMA-IR. Data are presented as the mean ± SD values and are representative of a single independent experiment (*n* = 6). Statistical analysis was performed using one-way ANOVA, followed by Tukey’s multiple comparison tests. ^a^Significantly different from the normal control group. ^b^Significantly different from the HFHF group. Differences were considered significant if the p-value was < 0.05. *CTRL* control, *ETA* etanercept, *HFHF* high-fat high-fructose diet, *HOMA-IR* hemostatic model assessment of insulin resistance, *IR* insulin resistance.

Compared with the normal control group, the HFHF group showed a two-fold increment in the fasting blood glucose level (Fig. 4a) and a five-fold augmentation in the serum insulin level (Fig. 4b). This induced a significant increase in the homeostasis model of assessment (HOMA) index by 19-fold (Fig. 4c). Treatment with ETA enhanced glucose utilization, with a significant decline in blood sugar and insulin levels by 61% and 69.7%, respectively, compared with the HFHF group. Consequently, the HOMA-IR was normalized (Fig. 4a–c, respectively).

### ETA-induced used biochemical changes

Preliminary biochemical examinations reveal that feeding rats with HFHF diet for 4 weeks showed a significant increase in total cholesterol (33.5%), triglycerides (TG; 253.3%), and fasting blood glucose (51.1%) levels in comparison with the normal control group (Table 1).

**Table 1:** Preliminary biochemical investigations after 4 weeks of HFHF diet.

Parameter	Control group	HFHF group
Cholesterol (mg/dL)	150.3 ± 4.1	200.8 ± 1.7^a^
TG (mg/dL)	52 ± 0.6	183.7 ± 3.6^a^
Fasting blood glucose (mg/dL)	60.1 ± 3.7	90.8 ± 0.2^a^

Data are presented as mean ± SD values and are representative of a single independent experiment (*n* = 4). Statistical analysis was performed using an unpaired t-test.

^a^Significantly different from the control group. Differences were considered significant if the p-value was < 0.05. *HFHF* high-fat high-fructose diet, *TG* triglycerides.

At the end of the experimental period (8 weeks), the HFHF group exhibited a significant increase in total cholesterol (180.6%), triglycerides (TG; 338.4%), and low-density lipoprotein (LDL; 334.7%) levels and a significant decline in high-density lipoprotein (HDL) level (71.2%) compared with the normal control group. This dyslipidemia induced by HFHF was efficiently alleviated by treatment with ETA, which restored total cholesterol, TG, and LDL to their normal levels. Further, ETA caused a significant elevation of HDL level by 93.5% compared with the HFHF group (Table 2).

**Table 2:** ETA reduces HFHF-induced elevation in serum liver function parameters and lipid profile.

Parameter	Control group	HFHF group	ETA-treated group
Cholesterol (mg/dL)	159.8 ± 4.7	448.9 ± 9.9^a^	166.8 ± 1.7^b^
TG (mg/dL)	50.0 ± 3.1	219.3 ± 10.1^a^	59.7 ± 1.2^b^
HDL (mg/dL)	78.4 ± 5.9	22.6 ± 2.8^a^	44.4 ± 1.0^ab^
LDL (mg/dL)	71.8 ± 2.3	312.8 ± 4.7^a^	75.2 ± 1.8^b^
ALT (U/L)	11.5 ± 2.4	87.6 ± 6.9^a^	18.3 ± 0.8^ab^
AST (U/L)	13.5 ± 2.9	98.4 ± 8.4^a^	23.5 ± 1.6^ab^

Data are presented as mean ± SD values and are representative of a single independent experiment (*n* = 6). Statistical analysis was performed using one-way ANOVA, followed by Tukey’s multiple comparison tests.

^a^Significantly different from the control group. ^b^Significantly different from the HFHF group. Differences were considered significant if the p-value was < 0.05. *ALT* alanine aminotransferase, *AST* aspartate aminotransferase, *ETA* etanercept, *HFHF* high-fat high-fructose diet, *HDL* high-density lipoprotein, *LDL* low-density lipoprotein, *TG* triglycerides.

Severe hepatic injury caused by the HFHF model was evaluated by estimating both serum alanine aminotransferase (ALT) and aspartate aminotransferase (AST) enzyme activities; in particular, both AST and ALT showed a significant elevation in their activities by six-fold (Table 2). Treatment with ETA significantly reduced AST and ALT activities by 76% and 79%, respectively, compared with the HFHF group (Table 2).

### ETA attenuated NASH by downregulating the hepatic TLR4 signaling pathway activated by the HFHF model

#### Hepatic TLR4 expression

**Figure 5:**
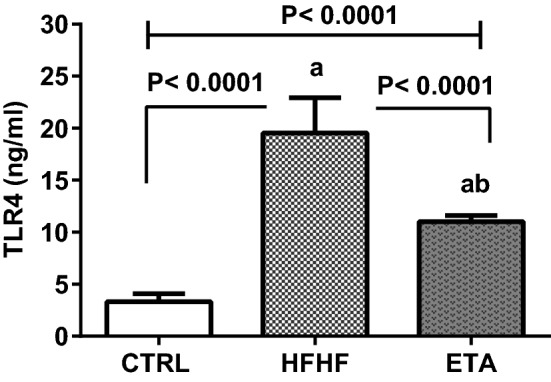
ETA reduces hepatic TLR4 concentrations. Comparison data from the HFHF-fed rats and standard chow-fed rats (CTRL) are shown. Data are presented as the mean ± SD values and are representative of a single independent experiment (*n* = 6). Statistical analysis was performed using one-way ANOVA, followed by Tukey’s multiple comparison tests. ^a^Significantly different from the normal control group. ^b^Significantly different from the HFHF group. Differences were considered significant if the p-value was < 0.05. *CTRL* control, *ETA* etanercept, *HFHF* high-fat high-fructose diet, *TLR4* Toll-like receptor 4.

Feeding rats with HFHF activated the hepatic TLR4 signaling pathway. Precisely, hepatic TLR4 concentrations showed an increment of 494% compared with the normal control group. Treatment with ETA significantly reduced hepatic TLR4 concentrations by 44% compared with the HFHF group (Fig. 5).

#### Immunohistochemical reactivity of NF-κB, TNF-α, and TGF-β1 observed in hepatic tissues

**Figure 6:**
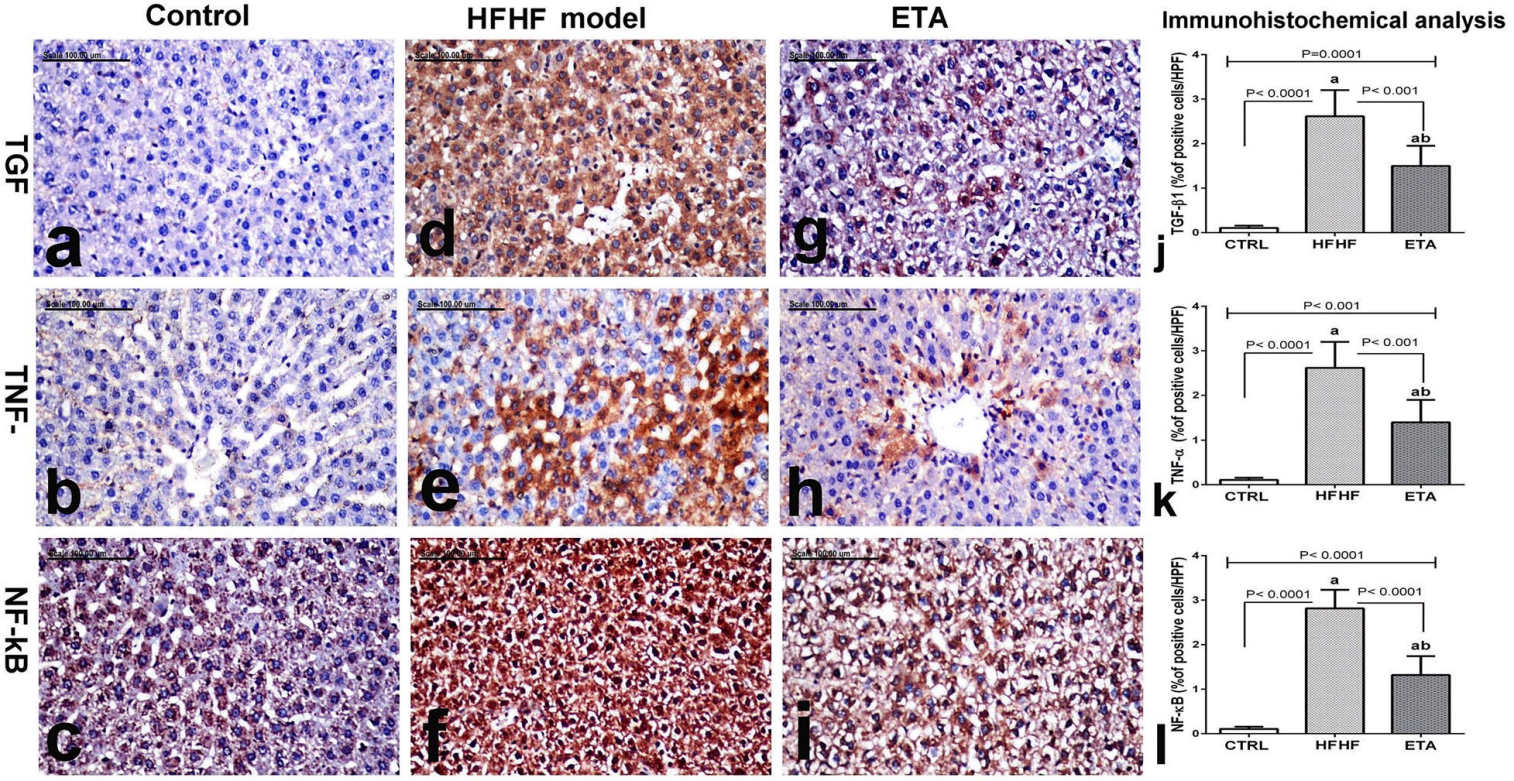
ETA attenuates NASH by downregulating the NF-κB/TNF-α/TGF-β1 signaling pathway activated by the HFHF model. Comparison data from the HFHF-fed rats and standard chow-fed rats (CTRL) are shown: immunohistochemically stained liver tissues of the normal control group (**a**–**c**) show no TGF-β1 (**a**) and TNF-α (**b**) expression and normal brown cytoplasmic staining of hepatocytes with no evidence of nuclear staining (**c**). The stained tissues of the HFHF group (**d**–**f**) show a significant increase of TGF-β1 (**d**) and TNF-α (**e**) positively stained cells and a significant increase of NF-κB positively stained cells with diffuse and extensive nuclear staining (**f**). The stained tissues of the ETA-treated group (**g**–**i**) show a few TGF-β1 (**g**) and TNF-α (**h**) positively stained cells along with sparse cells with brown nuclear staining (**i**). TGF-β1, TNF-α, and NF-κB immunohistochemical staining; scale bar = 100 µm. Data are presented as the mean ± SD values and are representative of a single independent experiment (*n* = 6). Statistical analysis was performed using one-way ANOVA, followed by Tukey’s multiple comparison tests. ^a^Significantly different from the normal control group. ^b^Significantly different from the HFHF group. Differences were considered significant if the p-value was < 0.05. *CTRL* control, *ETA* etanercept, *HFHF* high-fat high-fructose diet, *NF-κB* nuclear factor kappa beta, *TGF-β1* transforming growth factor beta, *TNF-α* tumor necrosis factor-alpha.

Immunohistochemical analysis results of TGF-β1, TNF-α, and NF-κB expression in hepatic tissues of all groups are illustrated in Fig. 6j–l, respectively. No TGF-β1 and TNF-α expression were demonstrated in hepatic tissues of control rats (Fig. 6a,b, respectively). Similarly, in the control group, NF-κB immunohistochemical analysis of the liver showed normal brown cytoplasmic staining of hepatocytes with no evidence of nuclear staining (Fig. 6c). By contrast, increased TGF-β1 and TNF-α expression with significant increase in the percentage of positively stained cells was observed in the hepatic tissues of the HFHF group (Fig. 6d,e, respectively). Additionally, a significant increase in NF-κB positively stained cells with diffuse and extensive nuclear staining was observed in the hepatic tissues of this group (Fig. 6f). By contrast, a significant decrease in TGF-β1, TNF-α, and NF-κB positively stained cells was noted in the ETA-treated group (Fig. 6g–i, respectively).

### Histopathological examination

**Figure 7:**
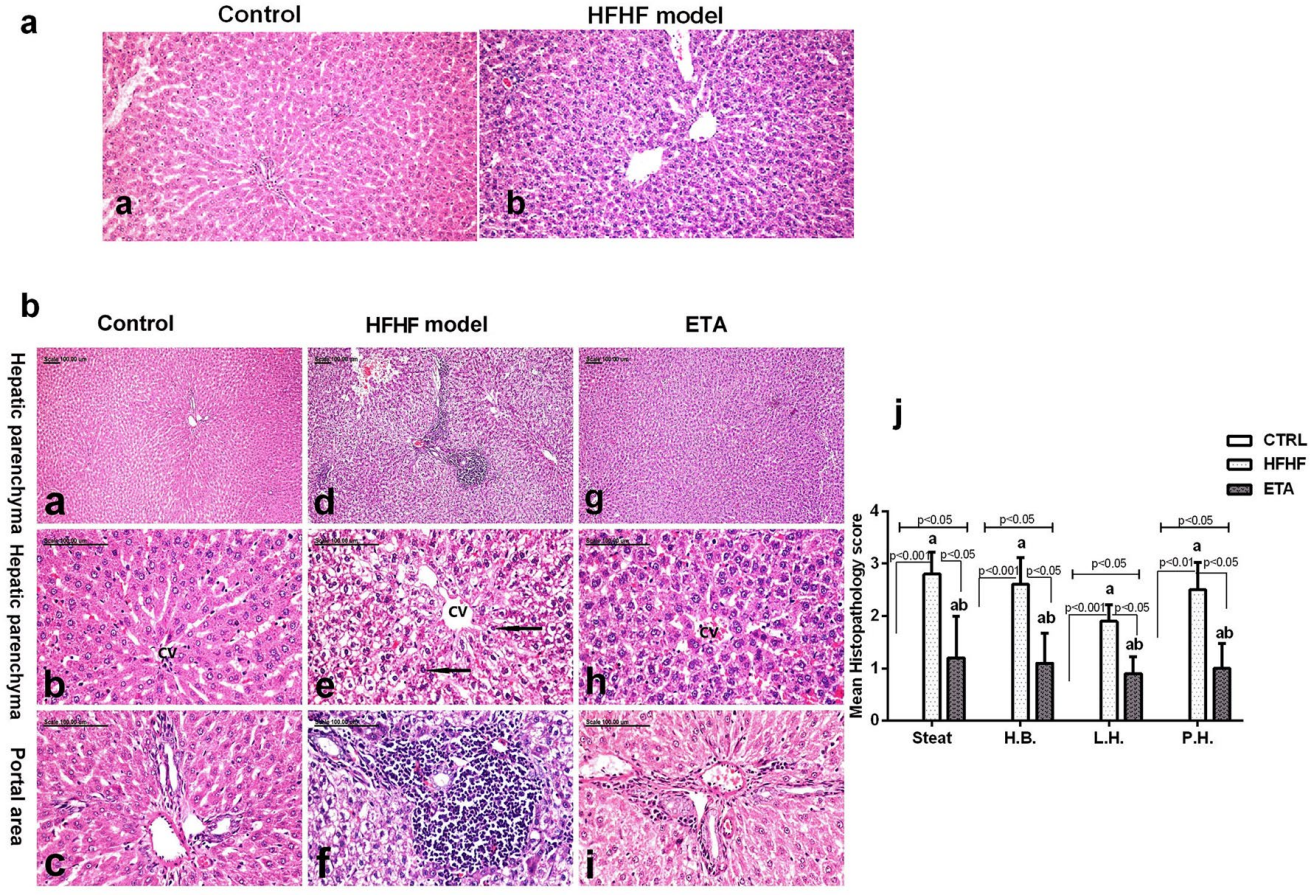
Histopathological changes in the hepatic tissues of rats using light microscopy. The photomicrographs show the following groups: In panel (**a**); The liver of normal control rats showed a standard histological structure with normal hepatocytes (**a**). The liver of HFHF-fed rats showed diffuse vacuolar degeneration of the hepatocytes which appeared greatly swollen (**b**). In panel (**b**); the normal control group (**a**–**c**) shows well-preserved hepatic parenchyma (**a**) with normal hepatocytes (**b**) and normal portal area (**c**), whereas the HFHF group (**d**–**f**) shows diffuse and extensive vacuolization of hepatocellular cytoplasm (**d**), with substantial swelling and ballooning of hepatocytes with fat globules and eccentric nuclei position (**e**) and intense infiltration of portal areas with mononuclear cells (**f**). The ETA-treated group (**g**–**i**) shows a marked reduction of hepatocellular swelling (**g** and **h**, respectively) and minimal infiltration of the portal area with few mononuclear cells (**i**). H&E stain; magnification, ×40; scale bar = 100 µm. (**j**) Mean pathological score recorded in all groups. Data are presented in the mean ± SD values and are representative of a single independent experiment (*n* = 6). Statistical analysis was performed using the Kruskal–Walli’s test, followed by Dunn’s post hoc test. ^a^Significantly different from the control group. ^b^Significantly different from the HFHF group. Differences were considered significant if the p-value was < 0.05. *CTRL* control, *ETA* etanercept, *HFHF* high-fat high-fructose diet, *HB* hepatocellular ballooning, *LH* lobular hepatitis, *PH* portal hepatitis.

Regarding panel (a) in Fig. 7, the preliminary histopathological examination that was carried out after 4 weeks of HFHF diet administration signified the emergence of NAFLD before starting ETA treatment. The liver of normal control rats showed a standard histological structure with normal hepatocytes (Fig. 7a). The liver of HFHF-fed rats showed diffuse vacuolar degeneration of the hepatocytes which appeared greatly swollen (Fig. 7b).

Regarding panel (b) in Fig. 7, the mean histopathological score estimated in the liver of the normal control and treated groups is shown in Fig. 7j. The liver of normal control rats showed well-preserved hepatic parenchyma with normal hepatocytes (Fig. 7a,b) and portal area (Fig. 7c). No inflammatory cellular infiltrates were demonstrated in the portal area of this group. By contrast, the liver of the HFHF group exhibited typical hepatic lesions of NASH, as it revealed diffuse and extensive vacuolization of hepatocellular cytoplasm (Fig. 7d) with marked swelling and ballooning of hepatocytes with fat globules and eccentric nuclei position (Fig. 7e). The portal areas were intensely infiltrated with mononuclear inflammatory cellular infiltrates (Fig. 7f). Other demonstrated lesions include lobular hepatitis, apoptotic figures, and focal hemorrhage. Pronounced attenuation of these histopathological lesions was demonstrated in the liver of the ETA-treated group, with a substantial reduction of hepatocellular swelling (Fig. 7g,h, respectively) and minimal infiltration of the portal area with few mononuclear cells (Fig. 7i). Moreover, apoptotic figures were demonstrated in this group.

**Figure 8:**
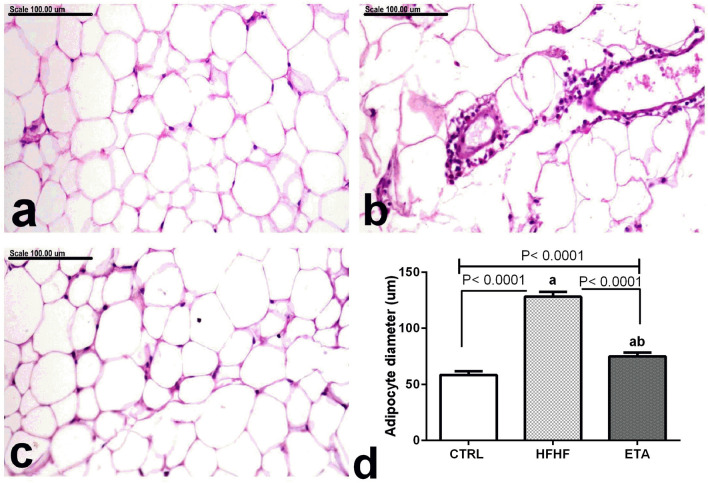
ETA diminished HFHF-induced changes in adipocyte size. The photomicrographs show abdominal adipose tissue of the following groups: (**a**) control rats showing normal unilocular white adipocytes, (**b**) HFHF model showing necrotic adipocytes surrounded by macrophages, (**c**) ETA-treated rats showing marked reduction of adipocytes size, (**d**) Mean of the adipocyte diameter measured in all groups. Data are the mean ± SD and are representative of a single independent experiment (*n* = 6). Statistical analysis was performed using one-way ANOVA, followed by Tukey’s multiple comparison tests. ^a^Significantly different from the control group. ^b^Significantly different from the HFHF group. Differences were considered statistically significant if p < 0.05. *CTRL* control, *ETA* etanercept, *HFHF* high-fat high fructose diet (stain: H&E, Scale bar = 100 µm).

The mean adipocyte diameter estimated in the abdominal adipose tissue of control rats and other treated groups is illustrated in (Fig. 8d). Visceral adipose tissue of control rats revealed normal white adipocytes with a mean diameter of 58.48 ± 1.38 µm (Fig. 8a). On the contrary, a remarkable increase of fat mass with pronounced expansion and hypertrophy of white adipocytes was recorded in the HFHF model, with a mean diameter of 128 ± 1.78 µm, which is significantly different from the control group. Moreover, numerous necrotic and/or apoptotic adipocytes surrounded by macrophages and lymphocytes were demonstrated in the HFHF model (Fig. 8b). Treatment with ETA revealed a significant decrease in adipocytes size, with a mean diameter of 75.12 ± 1.33 µm, concurrently with scant macrophages and lymphocytic cell infiltration (Fig. 8c).

## Materials and methods

### Animals and ethics approval

Animal handling procedures and experimental design were approved by the Faculty of Veterinary medicine, Cairo University Research Ethics Committee, Cairo, Egypt (Vet CU 2305 2022474). All experiments were performed in accordance with relevant guidelines and regulations and complied with the ARRIVE guidelines. Every effort was made to diminish the number of rats used and the distress caused to them in this study.

Adult male Wistar rats (140–150 g) were obtained from the animal house colony of the National Research Centre (NRC, Egypt) and acclimatized for 1 week before the experiments. Rats were housed in stainless-steel cages (3 rats per cage) that were kept under a controlled temperature of 24 °C ± 1 °C with a 12/12-h light–dark cycle (light cycle, 07:00–19:00). Rats were provided ad libitum access to a commercially available standard chow diet and water*.*

### Chemicals and antibodies

Fructose was purchased from El-Nasr Chemical Co. (Cairo, Egypt). ETA (Enbrel) was purchased from Wyeth (USA). All supplementary chemicals were of analytical grade. Polyclonal antibodies were specific to TNF-α (catalog no. sc-130220; 1:50 dilution; Santa Cruz Biotechnology, USA), NF-κB (catalog no. sc-109; 1:200 dilution, Santa Cruz Biotechnology), and TGF-β1 (catalog no. PA1-29020; 1:200 dilution; Thermo Fisher Scientific, Inc., USA).

### Experimental design

Animals were randomly distributed into three experimental groups (*n* = 6). All rats were provided with standard rodent chow and free access to drinking water. Rats in the group 1 were the normal control group. For effective induction of MS, rats in the group 2 (the HFHF group) and group 3 (the ETA-treated group) were administered high-fat diet (HFD) that was constructed according to Lasker, S. et al. who prepared it from normal rat feed, beef tallow, sucrose, and condensed milk, which represent ~ 14% proteins, 37% carbohydrates, and 49% fat from the perspective of the caloric content^9^, along with fructose solution dissolved in purified water (60%) intragastrically once daily^4^. From week 4 to the end of the 8-week experimental period, rats in group 3 received ETA (0.8 mg/kg twice weekly, subcutaneously)^14^.

For additional justification to specifically use this dose, by conversion of this dose to its counterpart in humans using Reagan-Shaw equations^29^ which described as follows:

$${\text{Human}}\,{\text{equivalent}}\,{\text{dose }}\left( {{\text{HED}}} \right) \, \left( {{\text{mg}}/{\text{kg}}} \right)\, = \,{\text{Animal}}\,{\text{dose }}\left( {{\text{mg}}/{\text{kg}}} \right){\text{ multiplied}}\,{\text{by}}\,{\text{animal}}\,{\text{Km}}/{\text{Human}}\,{\text{Km}}.$$

Given that: The dose of etanercept used in this study was 0.8 mg/kg twice per week, Km of rats is 6, and Km of humans is 37, An adult human weighs 60 kg will administrate 15.5 mg ETA per week. Considering the early human clinical trials, Phase II randomized, double-blind, placebo-controlled trials (RDBPCT) on patients with active RA used a dose ranging from 0.25 to 16 mg twice per week^30^. Therefore, this study will provide a safe and effective starting dose for clinical trials on metabolic syndrome.

Depending on the pilot study, the optimum treatment duration of ETA was adjusted.

Body weight was determined at 2-week intervals during the 8-week experimental period.

### Blood sampling, serum preparation, and tissue sampling

After 8 weeks, rats were fasted for 18 h to minimize feeding-induced variations in lipid patterns, and blood samples were collected from the retro-orbital sinus under light anesthesia. The blood samples were allowed to clot at a temperature of 25 °C, and serum was separated by centrifugation of the blood at 1409×*g* for 15 min using a centrifuge (Hettich Universal 32A, Germany). Each sample was divided into several aliquots, one for each of the biochemical parameters to be estimated for assessing the effect of ETA on the biochemical changes related to MS and stored at − 20 °C until analysis was performed.

Animals were euthanized by decapitation. A midline incision was made in the abdomen of all groups. The visceral adipose tissue, liver, and epididymal fat were carefully and rapidly excised. The isolated livers were washed with cold normal saline and dried on filter paper. The liver lobes were homogenized in ice-cold saline using a homogenizer (Heidolph Diax 900, Germany) to prepare a 20% homogenate. The prepared homogenate was divided into several aliquots and stored at − 20 °C until later use in assays for estimating the shortlisted biochemical parameters. The remaining part of the large hepatic lobe was fixed with 10% formaldehyde for histopathological examination.

### Histopathological and immunohistochemical examination

Different sections from liver tissues from all groups were excised and fixed in 10% buffered formalin. The tissues were routinely processed and embedded in paraffin blocks. Finally, the tissues were cut into 5-μm-thick sections and stained with hematoxylin and eosin (H&E) for conventional histopathological examination that was performed under light microscopy by a pathologist who was blinded to the therapeutic approach to diminish bias and variability. Images were captured using a Leica ICC50 HD digital camera attached to a Leica motorized light microscope system. To assess hepatocellular damage, lesion scoring was estimated in 10 random microscopic fields for each group. A semiquantitative lesion scoring system for NASH evaluation was performed according to Kleiner et al. and Mitchell et al.^31,32^, with some modifications. The description of this scoring system is illustrated in Table 3, which shows the scoring methodology for NASH assessment.

**Table 3:** NASH scoring system.

Steatosis grade		Lobular hepatitis (×20)	Hepatocellular ballooning (×10)	Portal hepatitis (×200)
0	< 5%	No foci	No ballooned hepatocytes	No foci
1	5–33%	< 2 foci per 20 × field	Few ballooned hepatocytes	< 2 foci per 200 × field
2	34–66%	2–4 foci per 20 × field	Several ballooned hepatocytes	2–4 foci per 200 × field
3	> 66%	> 4 foci per 20 × field	ND (not determined)	> 4 foci per 200 × field

In response to the histopathological analysis of visceral fat, the visceral adipose tissue was separated, fixed in 10% neutral formalin, and routinely processed. Then, 5-μm thick sections were cut into and stained with hematoxylin and eosin (H&E). According to the method of Patrick et al.^33^, evaluation of the histopathological alterations is demonstrated by the estimation of adipocyte diameter in ten random individual adipocytes.

All immunohistochemical procedures for the demonstration of TGF-β1, TNF-α, and NF-κB immunoreactivity were conducted according to Abd El-daim et al.^34^. Liver tissue sections (thickness of 5 μm) were dewaxed and rehydrated in graded alcohol. The tissues were incubated in 3% hydrogen peroxide to block the endogenous peroxidase activity. Subsequently, the sections were incubated with monoclonal anti-TNF-α, monoclonal anti-TGF-β1, and polyclonal anti-NF-κB antibodies. Finally, immunoreactivity was visualized using diaminobenzidine (Sigma, USA). Cells with brown cytoplasmic staining were considered positive for TGF-β1 and TNF-α, whereas cells with brown nuclear staining were considered positive for NF-κB. The assessment of TGF-β1, TNF-α, and NF-κB immunoreactivity was semi-quantitatively performed in 10 random high-power fields (HPFs) according to Ribeiro et al.^35^. A grading system scaled from 0 to 3 was used, relying on the percentage of positive cells in the microscopic HPF (40×) as follows: 0 = no staining, 1 = positive staining in < 30% of cells per HPF, 2 = positive staining in 30–70% of cells per HPF, and 3 = positive staining in > 70% of cells per HPF).

### Estimation of biochemical parameters

#### Serum adipokines

TNF-α, adiponectin, and leptin levels were estimated using ELISA Rat Immunoassay kits (Cusabio Biotech, USA; catalog nos. CSB-E11987r, CSB-E07271r, and CSB-E07433r, respectively) from R&D Systems, Inc., according to the manufacturer’s instructions.

#### Fasting blood glucose, serum insulin, and IR

Rats fasted overnight following the administration of the last dose of drugs. Fasting blood glucose was investigated in blood samples from the tail tip using an automatic blood glucose meter (Super Glucocard, ARKRAY, Japan).

Insulin levels were estimated using a Rat Insulin ELISA kit (Cusabio Biotech; catalog no. CSB-E05070r), according to the manufacturer’s instructions. IR was quantified using the HOMA formula: blood glucose (mg/dL) × serum insulin (U/mL)/405^36^.

#### Serum lipid profile and SBP

Serum LDL, HDL, total cholesterol, and TG were evaluated using colorimetric kits (Biodiagnostic, Egypt) via an ultraviolet–visible spectrophotometer (UV-1601PC; Shimadzu, Japan), according to the manufacturer’s instructions.

SBP was estimated by noninvasive tail-cuff plethysmography in conscious rats. An average of at least three readings per session was recorded. A pneumatic pulse transducer placed on the ventral surface of the tail, distal to the occlusion cuff, was used to identify the return of the pulse wave following a slow deflation of the cuff. Cuff pressure was obtained using a pneumatic pulse wave transducer via a programmed electro-sphygmomanometer PE-300 connected to a Physio-graph MK-IIIS for pulse recording (Narco Biosystems, Austin, TX, USA)^37^.

#### Liver function and hepatic TLR4 concentration

Serum AST and ALT levels were evaluated using colorimetric kits (Biodiagnostic, Egypt) according to Reitman and Frankl et al.^38^. Briefly, 2,4-dinitrophenylhydrazine (1 mmol/L) was added to the serum samples and incubated at 37 °C for 30 min. Absorbance was estimated (wavelength of 505 nm) with a double-beam spectrophotometer (Thermo Electron Corp., UK).

TLR4 concentrations were evaluated in liver tissues using an ELISA Rat Immunoassay kit (Cusabio Biotech; catalog no. CSB-E15822r) from R&D Systems, Inc., according to the manufacturer’s instructions.

### Statistical analysis

Data were presented as mean ± standard deviation (SD) values. Comparisons between mean values were made using one-way analysis of variance (ANOVA), followed by Tukey’s multiple comparison tests, except that the preliminary biochemical investigations were analyzed by using an unpaired t-test and the mean histopathological score was investigated by the Kruskal–Wallis test, followed by Dunn’s post hoc multiple comparison tests. For all statistical tests, the significance level was established at a p-value of < 0.05. GraphPad Prism^®^ software package version 6 (Graph Pad Software, Inc., USA) was used to perform all statistical tests.

## Discussion

The present study investigated the prospective therapeutic effects of ETA on HFHF-induced MS and its related complications in rats by counteracting the prominent features of MS.

The lack of a standard reference for MS model in rats that can induce all clinical manifestations and complications associated with MS as in humans stands as an obstacle during the preclinical studies of recent therapeutic medications^1^. To imitate MS in humans, rats in this study were fed with HFD concurrently with an administration of 60% fructose solution that stimulates the incidence and progression of metabolic disorders associated with MS for 8 weeks; this diet was compared with the administration of either high-fat diet only or fructose diet, which failed alone in this duration to present all metabolic features and complications associated with MS^4^.

This model presented the typical pathophysiology of MS, starting with a significant elevation in body weight, dyslipidemia, and IR until adipocyte dysfunction, which indicated the occurrence of authentic human MS^39^ and stimulated the incidence and progression of NAFLD-associated MS via the activation of the hepatic NF-κB/TNF-α/TGF-β1 signaling pathway.

In the present study, estimating the efficacy of ETA for the treatment of the prominent clinical features of MS and its related complications by observing its ability to attenuate the NF-κB/TNF-α/TGF-β1 signaling pathway activity and adipocyte dysfunction is a novel mode of action of ETA for the treatment of MS, compared with previous studies that have investigated TNF-α blockade in MS^25^.

Results showed a significant increase in the body weight of HFHF-fed rats compared with standard diet-fed rats in the normal control group, as shown previously^4^. This can be ascribed to the high intake of fats in the diet in addition to excessive consumption of fructose that stimulates the expression of lipogenic enzymes that induce obesity^2,9^. Taken together, upon HFD ingestion and high-fructose consumption, a state of chronic systemic inflammation developed in the body during which the TNF-α inflammatory mediator was secreted from adipocytes and inflammatory cells that are strongly related to obesity^41–43^.

These findings were consistent with the reports of Hsu et al., who observed that ETA administration significantly reduced HFD-induced obesity^14^. The ability of ETA to inhibit any increase in body weight is attributed to its inhibition of TNF-α, the key inducer of adipogenesis and obesity^42,44^.

As obesity evolves, adipocytes endure hypertrophy, owing to augmented TG storage, and a high percentage of adiposity causes metabolic dysfunction, with increased secretion of proinflammatory adipokines/cytokines, mainly leptin and TNF-α, but decreased secretion of anti-inflammatory adipokines, mainly adiponectin^45^.

Accordingly, marked hypoadiponectinemia and augmented TNF-α levels detected in the serum of the HFHF group contributed to stimulating the main clinical features of MS as IR and consequently hepatic steatosis^6,7^, which can be ascribed to adiponectin anti-inflammatory and anti-lipogenic effects^38^.

A potential correlation between obesity and hepatic leptin resistance noted by the elevated serum leptin levels in the HFHF group led to the failure of leptin to stimulate hepatic lipid turnover^45,46^; this failure induces IR and steatosis, which further develops hepatic fibrosis because leptin plays a proinflammatory role in mediating procollagen I, TGF-β1, and TNF-α expression^47^.

Previous studies have reported that TNF-α influences the adipose production of profibrogenic factors, such as leptin, and inhibits the expression and activity of adiponectin^47,48^. This resulted in significantly elevated adiponectin levels and normalized leptin levels in the ETA-treated group, which competitively prevented the binding of TNF-α to its receptor, causing its inhibition^14^. Consequently, ETA ameliorated adipocyte dysfunction and aids in the prevention of intrahepatic lipid accumulation and the enhancement of both insulin sensitivity^49^ and endothelial functions that counteract hypertension, as detected in the present study^27^.

One of the most prominent pathological features of MS correlated with the diagnostic conditions of human MS is IR^50^. In the present study, HFHF administration resulted in IR, as characterized by the elevated HOMA-IR score. Consequently, hyperinsulinemia-mediated impaired glucose utilization and hyperglycemia were observed in the HFHF group, consistent with previous studies^4,14,27^.

Lasker et al. reported that the administration of HFD for 8 weeks stimulates endotoxemia and inflammation, thus activating innate and adaptive immunity and inducing IR and hyperglycemia^9^. By contrast, the administration of high-fructose levels causes the downregulation of insulin receptors, with the impairment of insulin-stimulated glucose utilization and insulin sensitivity^2^. Moreover, the adipocyte dysfunction previously investigated in the HFHF group plays a role in IR development considering that adiponectin motivates AMP-activated protein kinase, thereby directly modulating glucose utilization and insulin sensitivity^2^. Notably, both IR and elevated leptin levels are strongly correlated to hypertension, which is indicated in the present study by significantly elevated SBP levels^2^.

The suppressing effect of TNF-α upon liver insulin action is achieved by interrupting insulin signaling via serine phosphorylation of insulin receptor proteins^50,51^. Accordingly, in the present study, the neutralization of TNF-α by ETA enhanced insulin sensitivity as indicated by the normalized HOMA-IR and significantly reduced fasting glucose and insulin serum levels compared with those in the HFHF group. This was in agreement with previous studies that concluded that ETA and other anti-TNF-α monoclonal antibodies promote gluconeogenesis inhibition by enhancing insulin signal transduction via the modulation of the TNF-α pathway^14^. Further, this finding could interpret how ETA ameliorated type 2 diabetes-associated MS^13^.

With adipocyte dysfunction, IR aids in peripheral adipose tissue lipolysis and fat flow to the liver, causing fatty liver, as it augments intrahepatic TG accumulation and inhibits fatty acid oxidation^23,52^. The histopathological observations in the present study are consistent with these studies and show a significant increase in the mean pathological score for hepatocellular ballooning and steatosis. These observations were confirmed by García-Berumen et al., who reported that harmful effects of high-fat content were aggravated by high-fructose consumption that displayed hepatic steatosis mimicking that observed in humans^53^.

In this study, the antisteatotic effect of ETA is shown by the improvement observed in the histopathological examination, with a significant decrease in the mean pathological score of steatosis and hepatocellular ballooning compared with the HFHF group, consistent with the literature^54^. This reliable influence of ETA is attributed to its potent activity in the TNF-α receptor blockade. TNF-α induces hepatic lipogenesis by increasing free fatty acid efflux to the liver and hepatic TG accumulation^14,54^. It is worth noting that, we observed during the study progress, that at the beginning of the study, Rats were suffering from hyperphagia and an increase in HFHF consumption. Contrary to the ETA-treated group which curbed this hyperphagic state and HFHF intake, this could be due to its potential effect on the enhancement of insulin^55^ and leptin sensitivity^56^. This can be considered as one of the main mechanisms by which ETA attenuates NAFLD.

The presence of high-fat content and a high-fructose concentration in the diet stimulates the overgrowth of gram-negative bacteria with their cell wall containing lipopolysaccharide (LPS) in the gut that further translocate from the gut owing to impairments in the intestinal barrier function, causing endotoxemia and inflammation. Increased endotoxins accelerate the progression of hepatic inflammation and fibrosis in animal models via the interaction of bacterial fragments with TLR4^9,10,57^.

In Kupffer cells, the TLR4/NF-κB/TNF-α/TGF-β1 signaling pathway is essential for the progression of NAFLD to NASH^58^. The binding of circulating LPS with TLR4 activates NF-κB downstream signaling, thereby triggering the production of TNF-α, the crucial proinflammatory cytokine in NASH pathogenesis^10,58,59^. Consequently, the activation of the TLR4 signaling pathway sensitizes hepatic stellate cells, leading to the evolvement of the profibrogenic signaling molecule TGF-β1 that initiates hepatic fibrosis^58^. Notably, feeding rats with the HFHF diet caused endotoxemia, as evidenced by the significant activation of hepatic TLR4, followed by NF-κB and TNF-α overexpression, compared with the normal control group; this observation indicated that the HFHF model effectively targeted the major inflammatory pathway that induces NASH-associated MS. Taken together, TGF-β1 overexpression along with augmented leptin levels observed in the model group evidenced the occurrence of early fibrotic steatohepatitis, according to the literature^4,10,60^.

In the current study, compared with the HFHF group, the ETA-treated group showed a significant reduction in TNF-α levels. The anti-inflammatory effect exerted by ETA is attributed to its structure as a soluble TNF p75 receptor^61^. Although ETA mainly acts on TNF-α and prevents interaction with its receptor, this study demonstrated suppression in hepatic TLR4 activity. Expounding this result, Fossati et al. concluded that TNF-α monoclonal antibodies could potently inhibit the LPS-driven cytokine production and dramatically reduce the cell surface protein and mRNA levels of TLR4 activated with LPS, as it reduced their capacity to bind to TLR4 and transfer the inflammatory signal into the cell^62^. This is consistent with the findings of Yang et al. who stated that ETA sharply downregulates TLR4 expression^63^.

Passing through this signaling pathway, the immunohistochemical findings revealed that ETA translocated NF-κB, a downstream molecule of TLR4, to the nucleoli and disabled the progression of NASH to fibrosis via TGF-β1 suppression. These data were consistent with those obtained by Yao et al. who reported the suppression of NF-κB under the influence of ETA^64^.

In the HFHF group, an evident increase in ALT and AST parameters was observed owing to the dramatic hepatic damage prompted by the combined administration of the HFHF diet, thereby disrupting the functional integrity of the hepatic membrane and consequently leading to the leakage of cellular enzymes^4,10^. The reduction in ALT and AST enzyme activities observed in the ETA-treated group confirmed that ETA caused hepatic regeneration and preserved its functional integrity^65^.

In the present study, serum TG, total cholesterol, and LDL levels in HFHF-fed rats showed significant elevation, whereas HDL levels were prominently reduced compared with those in the normal control group. The induction of dyslipidemia in response to HFHF administration agreed with previous studies^2,4,9^.

By contrast, the significant improvement of serum lipid profile parameters observed in the ETA-treated group was relevant to its potent TNF-α inhibiting effect besides its effect on leptin and adiponectin, as evidenced by Zuo et al. who reported that a high adiponectin/leptin ratio is associated with lower plasma TG, total cholesterol, and higher HDL levels^66^. This finding was consistent with Hsu et al. who confirmed the positive effect of ETA on dyslipidemia^14^.

## Conclusion

The present study proposed that the blockade of the TNF-α inflammatory pathway by ETA has a curable effect on rats with HFHF-induced MS. Treatment with ETA showed an enhancement of all typical features related to MS, as ETA combats obesity in rats and decreases the elevated serum adipokine level and hypertension. Moreover, ETA exerted significant beneficial differences in glucose homeostasis, dyslipidemia, and liver function tests as well as significantly ameliorated NASH and even MS-associated early hepatic fibrosis via the attenuation of the NF-κB/TNF-α/TGF-β1 signaling pathway in the liver; this pathway was activated in HFHF-fed rats. Therefore, treating MS and its related complications using ETA, an anti-TNF-α monoclonal antibody, appears to be an effective therapeutic approach for this indication that could be developed after further investigations on human subjects in clinical trials.

## Data Availability

The data generated during and /or analyzed during the current study are available from the corresponding author upon reasonable request.

## References

[1] Gunawan S, Aulia A, Soetikno V (2021). Development of rat metabolic syndrome models: A review. Vet. World..

[2] Yahia H, Hassan A, El-Ansary MR, Al-Shorbagy MY, El-Yamany MF (2020). IL-6/STAT3 and adipokine modulation using tocilizumab in rats with fructose-induced metabolic syndrome. Naunyn. Schmiedebergs. Arch. Pharmacol..

[3] Rodríguez-Correa E, González-Pérez I, Clavel-Pérez PI, Contreras-Vargas Y, Carvajal K (2020). Biochemical and nutritional overview of diet-induced metabolic syndrome models in rats: What is the best choice?. Nutr. Diabetes.

[4] LoredoMendoza MBJ (2014). A quick model for the induction of metabolic syndrome markers in rats. Intern. Med. Open Access.

[5] Rizkalla SW (2010). Health implications of fructose consumption: A review of recent data. Nutr. Metab..

[6] Matsumoto H (2006). Adiponectin deficiency exacerbates lipopolysaccharide/D-galactosamine-induced liver injury in mice. World J. Gastroenterol..

[7] Furukawa S (2017). Increased oxidative stress in obesity and its impact on metabolic syndrome. Find the latest version: Increased oxidative stress in obesity and its impact on metabolic syndrome. J. Clin. Investig..

[8] Chan AML (2021). Recent developments in rodent models of high-fructose diet-induced metabolic syndrome: A systematic review. Nutrients.

[9] Lasker S (2019). High-fat diet-induced metabolic syndrome and oxidative stress in obese rats are ameliorated by yogurt supplementation. Sci. Rep..

[10] Liu J (2014). Toll-like receptor-4 signalling in the progression of non-alcoholic fatty liver disease induced by high-fat and high-fructose diet in mice. Clin. Exp. Pharmacol. Physiol..

[11] Danila, M. I., Hughes, L. B. & Bridges, S. L. Pharmacogenetics of etanercept in rheumatoid arthritis. *Pharmacogenomics* **9**, (2008). 10.2217/14622416.9.8.1011 PMC3746504 18681777

[12] Waters JP, Pober JS, Bradley JR (2013). Tumour necrosis factor and cancer. J. Pathol..

[13] Dik B, Bachivan E, Eser Faki H, Uney K (2018). Combined treatment with interlukin-1 and tumor necrosis factor-alpha antagonists improve type 2 diabetes in rats Burak DIK*, Emre BAHCIVAN, Hatice ESER FAKI, Kamil UNEY. Can. J. Physiol. Pharmacol..

[14] Hsu CC (2021). Etanercept ameliorates cardiac fibrosis in rats with diet-induced obesity. Pharmaceuticals.

[15] Anker SD, Coats AJS (2002). How to RECOVER from RENAISSANCE? The significance of the results of RECOVER, RENAISSANCE, RENEWAL and ATTACH. Int. J. Cardiol..

[16] Pfeifer EC, Saxon DR, Janson RW (2017). Etanercept-induced hypoglycemia in a patient with psoriatic arthritis and diabetes. J. Investig. Med. High Impact Case Rep..

[17] Al-Mutairi N, Shaaban D (2016). Effects of tumor necrosis factor α inhibitors extend beyond psoriasis: Insulin sensitivity in psoriasis patients with type 2 diabetes mellitus. Cutis.

[18] Sakunrangsit N (2021). Etanercept prevents TNF-α mediated mandibular bone loss in FcγRIIb−/− lupus model. PLoS One.

[19] Agca R, Heslinga M, Kneepkens EL, Van Dongen C, Nurmohamed MT (2017). The effects of 5-year etanercept therapy on cardiovascular risk factors in patients with psoriatic arthritis. J. Rheumatol..

[20] Dominguez H (2005). Metabolic and vascular effects of tumor necrosis factor-α blockade with etanercept in obese patients with type 2 diabetes. J. Vasc. Res..

[21] Clark IA (2020). Randomized controlled trial validating the use of perispinal etanercept to reduce post-stroke disability has wide-ranging implications. Expert Rev. Neurother..

[22] Bernstein LE, Berry J, Kim S, Canavan B, Grinspoon SK (2006). Effects of etanercept in patients with the metabolic syndrome. Arch. Intern. Med..

[23] Velikova, T. V., Kabakchieva, P. P., Assyov, Y. S. & Georgiev, T. A. Targeting inflammatory cytokines to improve type 2 diabetes control. *Biomed. Res. Int.* **2021**, (2021). 10.1155/2021/7297419 PMC8455209 34557550

[24] Mascolo A (2022). Current and future therapeutic perspective in chronic heart failure. Pharmacol. Res..

[25] Qian, Y., Mao, M. & Nian, F. The effect of TNF-α on CHD and the relationship between TNF-α antagonist and CHD in rheumatoid arthritis: A systematic review. **2022**, (2022). 10.1155/2022/6192053 PMC9433296 36060429

[26] Răzvan-Valentin S (2022). Etanercept prevents endothelial dysfunction in cafeteria diet-fed rats. Int. J. Environ. Res. Public Health.

[27] Tran LT, MacLeod KM, McNeill JH (2009). Chronic etanercept treatment prevents the development of hypertension in fructose-fed rats. Mol. Cell. Biochem..

[28] Hafez, H. M. *et al. Potential Protective Effect of Etanercept and Aminoguanidine in Methotrexate-Induced Hepatotoxicity and Nephrotoxicity in Rats*, vol. 768 (Elsevier, 2015). 10.1016/j.ejphar.2015.08.047 26332135

[29] Reagan-Shaw S, Nihal M, Ahmad N (2008). Dose translation from animal to human studies revisited. FASEB J..

[30] Fleischmann R, Stern R, Iqbal I (2004). Etanercept—Review of efficacy and safety after five years of clinical use. Therapy.

[31] Mitchell JR (1973). Acetaminophen induced hepatic necrosis. I. Role of drug metabolism. J. Pharmacol. Exp. Ther..

[32] Kleiner DE (2005). Design and validation of a histological scoring system for nonalcoholic fatty liver disease. Hepatology.

[33] Morris PG (2011). Breast tissue of obese women with breast cancer. Cancer Prev. Res. (Phila).

[34] Abd Eldaim MA, Ibrahim FM, Orabi SH, Hassan A, El Sabagh HS (2018). L-Carnitine-induced amelioration of HFD-induced hepatic dysfunction is accompanied by a reduction in hepatic TNF-α and TGF-β1. Biochem. Cell Biol..

[35] Ribeiro PS (2004). Hepatocyte apoptosis, expression of death receptors, and activation of NF-κB in the liver of nonalcoholic and alcoholic steatohepatitis patients. Am. J. Gastroenterol..

[36] Matthews DR (1985). Homeostasis model assessment: Insulin resistance and β-cell function from fasting plasma glucose and insulin concentrations in man. Diabetologia.

[37] Otero-Losada, M. *et al.* Cardiorenal Involvement in Metabolic Syndrome Induced by Cola Drinking in Rats: Proinflammatory Cytokines and Impaired Antioxidative Protection. *Mediat. Inflamm.* **2016**, (2016). 10.1155/2016/5613056 PMC4906210 27340342

[38] Reitman S, Frankel S (1957). A colorimetric method for the determination of serum glutamic oxalacetic and glutamic pyruvic transaminases. Am. J. Clin. Pathol..

[39] Zou Y (2006). High-fat emulsion-induced rat model of nonalcoholic steatohepatitis. Life Sci..

[40] Abdelhamid YA, Elyamany MF, Al-Shorbagy MY, Badary OA (2021). Effects of TNF-α antagonist infliximab on fructose-induced metabolic syndrome in rats. Hum. Exp. Toxicol..

[41] Stanley TL (2011). TNF-α antagonism with etanercept decreases glucose and increases the proportion of high molecular weight adiponectin in obese subjects with features of the metabolic syndrome. J. Clin. Endocrinol. Metab..

[42] Xiao QZ, Zhu LJ, Fu ZY, Guo XR, Chi X (2020). Obesity related microRNA-424 is regulated by TNF-α in adipocytes. Mol. Med. Rep..

[43] Kelany ME, Hakami TM, Omar AH (2017). Curcumin improves the metabolic syndrome in high-fructosediet-fed rats: Role of TNF-α, NF-κB, and oxidative stress. Can. J. Physiol. Pharmacol..

[44] Duan Y (2018). Inflammatory links between high fat diets and diseases. Front. Immunol..

[45] Poret JM (2018). High fat diet consumption differentially affects adipose tissue inflammation and adipocyte size in obesity-prone and obesity-resistant rats. Int. J. Obes..

[46] Issa D, Patel V, Sanyal AJ (2018). Future therapy for non-alcoholic fatty liver disease. Liver Int..

[47] Ikejima K (2001). Leptin augments inflammatory and profibrogenic responses in the murine liver induced by hepatotoxic chemicals. Hepatology.

[48] Stojsavljević S, Gomerčić Palčić M, Virović Jukić L, Smirčić Duvnjak L, Duvnjak M (2014). Adipokines and proinflammatory cytokines, the key mediators in the pathogenesis of nonalcoholic fatty liver disease. World J. Gastroenterol..

[49] Calamita G, Portincasa P (2007). Present and future therapeutic strategies in non-alcoholic fatty liver disease. Expert Opin. Ther. Targets..

[50] Farag MM, Ashour EH, El-Hadidy WF (2020). Amelioration of high fructose diet-induced insulin resistance, hyperuricemia, and liver oxidative stress by combined use of selective agonists of PPAR-α and PPAR-γ in rats. Dubai Med. J..

[51] Niederreiter L, Tilg H (2018). Cytokines and fatty liver diseases. Liver Res..

[52] Orlik, B., Handzlik, G. & Olszanecka-Glinianowicz, M. Rola adipokin i insulinooporności w patogenezie niealkoholowej stłuszczeniowej choroby wątroby The role of adipokines and insulin resistance in the pathogenesis of nonalcoholic fatty liver disease. *Postęp Hig. i Med. Doświadczalnej* **64**, (2010). 20498498

[53] García-Berumen CI (2019). The severity of rat liver injury by fructose and high fat depends on the degree of respiratory dysfunction and oxidative stress induced in mitochondria. Lipids Health Dis..

[54] Yalcin M (2014). A comparison of the effects of infliximab, adalimumab, and pentoxifylline on rats with non-alcoholic steatohepatitis. Turk. J. Gastroenterol..

[55] Stagakis I (2012). Anti-tumor necrosis factor therapy improves insulin resistance, beta cell function and insulin signaling in active rheumatoid arthritis patients with high insulin resistance. Arthritis Res. Ther..

[56] Gaetke LM, Oz HS, McClain CJ, Frederich RC (2003). Anti-TNF-α antibody normalizes serum Leptin in IL-2 deficient mice. J. Am. Coll. Nutr..

[57] Margoni A (2011). Serum leptin, adiponectin and tumor necrosis factor-α in hyperlipidemic rats with/without concomitant diabetes mellitus. Mol. Med..

[58] Miura K, Ohnishi H (2014). Role of gut microbiota and Toll-like receptors in nonalcoholic fatty liver disease. World J. Gastroenterol..

[59] Lin X (2015). Role of APN and TNF-α in type 2 diabetes mellitus complicated by nonalcoholic fatty liver disease. Genet. Mol. Res..

[60] Browning JD, Horton JD (2004). Molecular mediators of hepatic steatosis and liver injury. J. Clin. Investig..

[61] Sakunrangsit N (2021). Etanercept prevents TNF-α mediated mandibular bone loss in FcγRIIb−/− lupus model. PLoS ONE.

[62] Fossati G, Nesbitt A (2008). The levels of TLR2, TLR4 and CD14 on LPS stimulated monocytes are reduced by membrane TNF signaling of certolizumab pegol, adalimumab and infliximab. Inflamm. Bowel Dis..

[63] Yang M, Chen J, Zhao J, Meng M (2014). Etanercept attenuates myocardial ischemia/reperfusion injury by decreasing inflammation and oxidative stress. PLoS ONE.

[64] Yao Y (2021). Etanercept as a TNF-alpha inhibitor depresses experimental retinal neovascularization. Graefe’s Arch. Clin. Exp. Ophthalmol..

[65] Kronborg, T. M., Ytting, H., Hobolth, L., Møller, S. & Kimer, N. Novel Anti-inflammatory Treatments in Cirrhosis. A Literature-Based Study. *Front. Med.* **8**, (2021). 10.3389/fmed.2021.718896 PMC8495012 34631742

[66] Zuo H (2013). Association between serum leptin concentrations and insulin resistance: A population-based study from China. PLoS ONE.

